# The Effects of Gender on Mesenchymal Stromal Cell (MSC) Proliferation and Differentiation In Vitro: A Systematic Review

**DOI:** 10.3390/ijms252413585

**Published:** 2024-12-19

**Authors:** Antonia Vogt, Anissa Faher, Joanna Kucharczak, Mark Birch, Andrew McCaskie, Wasim Khan

**Affiliations:** 1 Division of Trauma & Orthopaedic Surgery, Addenbrooke’s Hospital, University of Cambridge, Cambridge CB2 0QQ, UK; av591@cam.ac.uk (A.V.);; 2 School of Clinical Medicine, University of Cambridge, Cambridge CB2 2SP, UK

**Keywords:** differentiation, characterization, proliferation, gender, MSCs

## Abstract

Mesenchymal stromal cells (MSCs) have the potential for novel treatments of several musculoskeletal conditions due to their ability to differentiate into several cell lineages including chondrocytes, adipocytes and osteocytes. Researchers are exploring whether this could be utilized for novel therapies for joint afflictions. The role of gender in the ability of MSCs to differentiate and proliferate into different cells has not been clearly defined. This systematic review aims to report the current literature on studies, characterized by high quality and in-depth analysis even though quantitatively limited, that have looked at the role of gender in the differentiation and proliferation of MSCs. Sixteen studies were identified during the literature search, reporting 533 patients, of which 202 were male and 331 were female. MSC proliferation, phenotypic analysis and differentiation are reported and contrasted in terms of donor gender. Heterogeneity in methodologies across studies likely contributes to the inconclusive findings presented here, with no discernible statistical disparity observed between genders in differentiation traits. Nevertheless, the proliferation results indicate a notable gender-related impact. Future investigations should aim to ascertain the potential influence of gender on MSC proliferation capacities more conclusively, emphasizing the necessity of standardized protocols for MSC analyses to enhance accuracy and comparability across studies.

## 1. Introduction

Mesenchymal stromal cells (MSCs) demonstrate promising and novel pathways in many musculoskeletal (MSK) diseases and injury states and have been the source of active research. Degenerative diseases and trauma to joints have focused on lifestyle changes and pharmacological and surgical options to treat these particular conditions [1,2]. MSCs provide a novel pathway in cellular differentiation and healing that has allowed researchers to interrogate and assess if they work in particular MSK disease states [3,4].

MSCs have the potential to differentiate into different mesoderm-derived specialized cells, which include osteoblasts, chondrocytes, adipocytes and tenocytes, and therefore, its potential multicellular properties allow it to potentially change the course of the disease state in question [5,6]. Osteoarthritis occurs as a disease or injury with poor healing and changes in the joint’s architecture, including ligamentous and tendinous injuries [7,8]. With osteoarthritis, the predominant issue is one of cartilaginous loss with time or as a result of prior trauma to the joint. It is thought that MSCs may also function using immunomodulation, whereby they influence the reparative process within a joint [9]. With osteoarthritis, this works by activating chondrocyte differentiation with the potential to lay down new cartilage. Previous research has demonstrated that MSCs are capable of generating the primary components of articular cartilage: (1) proteoglycans, which impart compressive stiffness to the cartilage, and (2) type II collagen, which enhances the cartilage’s tensile strength and resilience [10].

During soft tissue injury of joints, particularly with ligaments and tendons, the healing process remains long [11]. This is partly a result of the poor vasculature in these areas; therefore, patients are immobilized for several months to allow the body to heal these structures. Studies show that these areas, although able to heal, do not always appear to have the same tensile strength as an uninjured tendon/ligament and therefore are at higher risk of re-rupture [12]. MSCs have the potential to both speed up this healing process but also allow stronger healing of these regions through actions that may include immunomodulation of cells during the active phase of the injury, angiogenesis within the region and the differentiation of tenocytes to allow the appropriate extracellular matrix and cell deposition to restore the tensile strength of these regions [13,14].

Many of the studies in the literature have shown MSC potential in vitro, but not all studies have looked at this in vivo. In particular, it is unclear whether gender plays a role in the ability of MSCs to differentiate appropriately within these injury or disease states or whether gender and MSC use differently influence the homeostatic and reparative mechanisms associated with reported immunomodulation that occurs with the use of MSCs [15]. Women, for example, are more likely to develop osteoarthritis following menopause where there is an accelerated state of bone metabolism as a result of the loss of estrogen in normal bone homeostasis [16,17,18,19,20]. Whether this endocrine function influences the MSC state within the joint remains to be seen and is something that remains unknown in the literature.

### Aim

This systematic review aimed to identify and report on whether the gender of a patient affected the proliferation and differentiation capacity within mesenchymal stromal cells. The primary goal was to report from the available literature and studies to date as to whether gender influences this capacity and to amalgamate this together.

## 2. Materials and Methods

### 2.1. Search Strategy

A systematic review of the literature was conducted following the guidelines outlined in the Preferred Reporting Items for Systematic Reviews (PRISMA) [21,22]. The literature search spanned four major databases: PubMed, Embase, Medline, and SCOPUS. Searches were executed during the last week of October and the first week of November. The strategy employed specific search terms, as detailed below: “sex” or “gender” and “mesenchymal stem cells” or “mesenchymal stem cell” or” mesenchymal stromal cells” or “mesenchymal stromal cell” and “cell surface characterisation” or “cell surface” or “differentiation potential” or “differentiation” and “in vitro”.

**Figure 1:**
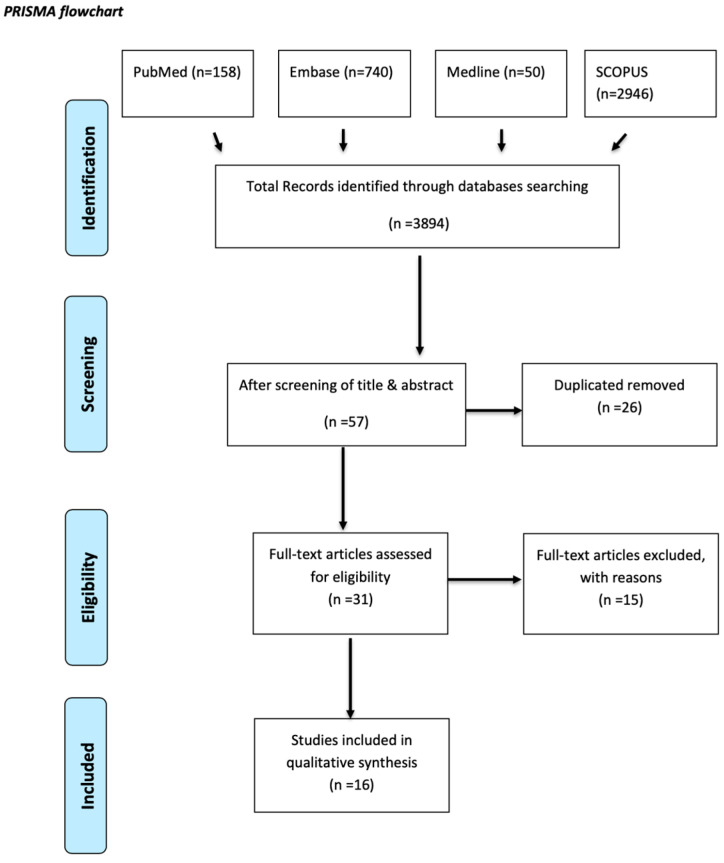
PRISMA flow diagram [21]. n = number of records.

One hundred and fifty-eight studies were extracted from PubMed (1946—last week of August 2024), 740 from Embase (1996—last week of August 2024), 50 from Medline (1946—first week of August 2024), and 2946 from SCOPUS (1900—last week of August 2024). In total, 3894 papers were identified. After careful screening of titles and abstracts, we narrowed down our selection to 57 papers. A total of 26 duplicate papers were identified and excluded from the analysis. After applying the inclusion and exclusion criteria, the remaining studies were screened based on their titles and abstracts. Subsequently, 31 studies were reviewed in full but excluded according to the exclusion criteria outlined below. This process, detailed in Figure 1, resulted in the identification of 16 papers for data extraction. The search was carried out by (AV, AF, and JK), with two authors (WK and AV) independently reviewing the titles and abstracts. Disagreements were resolved by including the studies for a full-text review.

The study was registered on the PROSPERO database with the registration number (247882), and the protocol can be accessed at the following web address.

### 2.2. Inclusion Criteria

1. In vitro studies involving adult human subjects;
2. Studies with a reference to subjects’ gender/sex;
3. Studies looking at MSCs and with source of extraction of cells specified;
4. Studies that investigated at least one lineage of differentiation or MSC characterization;
5. English language.

### 2.3. Exclusion Criteria

1. Duplicate studies;
2. Those not in the English language;
3. All non-human in vitro or in vivo studies;
4. Studies using samples from patients with multisystem diseases and malignancies;
5. Any paper other than research papers was excluded;
6. Studies looking at non-mesenchymal cells, e.g., embryonic, umbilical cord and periodontal MSCs.

### 2.4. Data Extraction

Data were extracted and organized using an Excel spreadsheet for systematic analysis. The extracted information is summarized in four tables, which include the reference details, a concise description of the study, the number and gender of patient donors, the source of the MSCs, culture conditions, proliferation data, MSC surface markers characterization, and results from chondrogenic, adipogenic, and osteogenic differentiation analyses.

### 2.5. Quality Assessment

The quality of each included study was evaluated using a modified version of the “OHAT Risk of Bias Rating Tool for Human and Animal Studies” developed by the Office of Health Assessment and Translation (OHAT) [23]. Any discrepancies in the quality assessment results were resolved through discussion and consensus among the authors.

## 3. Results

### 3.1. General Characteristics of Included Studies

This systematic review included a total of 16 studies, as illustrated in Figure 1. The studies that met the inclusion and exclusion criteria ranged from the earliest, published in 2008 [24], to the most recent, published in 2023 [25]. In the majority of the included studies, MSCs were extracted from various tissues of both healthy individuals and osteoarthritic patients, and the proliferation of different MSC types was assessed.

All studies included investigated the gender effect on adult MSCs derived from different sources of the human body; however, non-MSCs like embryonic, umbilical cord and periodontal stem cells were excluded. Periodontal cells were not included in this systematic review due to their distinct developmental origin and functional properties compared to other MSCs. Firstly, periodontal stem cells exhibit unique developmental origins compared to other MSCs, being derived from the ectoderm rather than the mesoderm like most MSCs found below the neck. This difference in embryonic origin suggests that periodontal cells may possess distinct developmental memory and functional characteristics, potentially leading to different responses to gender-related factors. Secondly, the focus of the systematic review was likely on MSCs derived from tissues with similar developmental origins to ensure a more homogeneous analysis of gender effects. Thirdly, the specific differentiation pathways and functional properties of periodontal cells, which are primarily involved in the formation of periodontal ligaments and alveolar bone, may not align with the broader scope of the review, which likely centered on the regenerative potential and therapeutic applications of MSCs. Therefore, to maintain consistency and relevance in the analysis of gender effects on mesenchymal stromal cells, periodontal cells were likely excluded from the review.

Sixteen papers were identified following a literature search with the aforementioned search criteria outlined in the methodology (Table 1). Of these, several studies looked at either chondrogenic or osteogenic potential in different sex groups. A total of seven papers looked at chondrocyte differentiation capacity between age groups, nine investigated the impact of sex on osteogenic potential, and five papers looked at adipogenic differentiation variability between the sexes. Three papers focused purely on human muscle progenitor cells and one paper looked at perivascular differentiation. The donor sites of these cells varied in each of the studies with a predilection to knee and synovial tissue to extract MSCs and the analysis of these cell characteristics was performed ex vivo. A total number of 533 patients (range 6–131 patients) were recruited into the 15 studies with a gender distribution of 202 males to 331 females (ratio 1:1.6, M:F). One study did not report the total number of patients or sex ratio [26].

Each of these studies used slight variations and differing methodologies to isolate and positively identify cells through a culmination of immunohistochemistry, genetic analysis and biochemical studies. These are reported in Table 1, Table 2, Table 3 and Table 4.

### 3.2. Gender Effect

**Figure 2:**
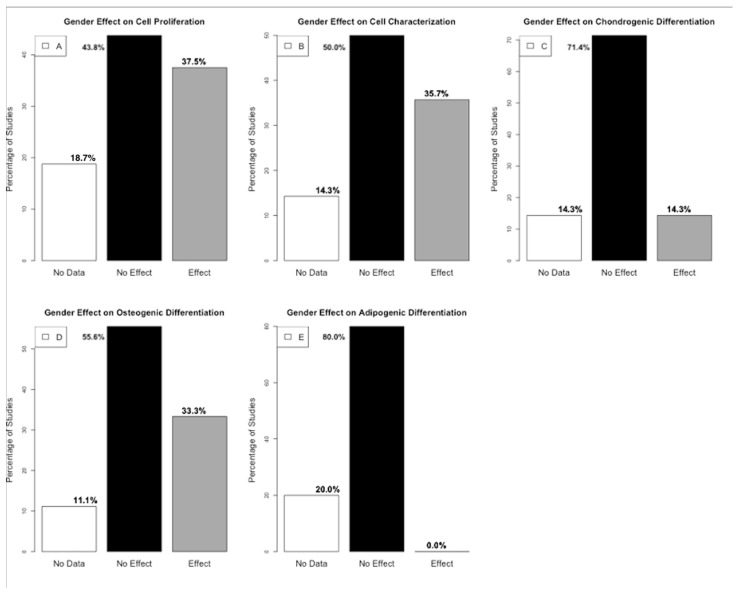
(**A**). Histogram presenting the percentage distribution of gender effect out of the 16 studies on cell proliferation. (**B**). Histogram presenting the percentage distribution of gender effect out of the 14 studies on cell characterization (**C**). Histogram presenting the percentage distribution of gender effect out of the seven studies on chondrogenic differentiation. (**D**). Histogram presenting the percentage distribution of gender effect out of the nine studies on osteogenic differentiation. (**E**). Histogram presenting the percentage distribution of gender effect out of the five studies on adipogenic differentiation (generated by R version 4.4.0).

A summary of the presence or absence of gender-related effects on cell proliferation, characterization, and differentiation, based on statistical outcomes was analyzed (Figure 2). The boundary distinguishing between “effect” and “no effect” was established through observations conducted over the same period, specifically evaluating differences between genders. Key parameters assessed included gene expression and cell count, among others.

### 3.3. Proliferation Analysis

Proliferation analysis between the different studies used a combination of immunohistochemistry, flow cytometry, genetic analysis and biochemical assays as well as micro-CT to report the difference in population numbers between the sexes. In summary, all 16 studies followed a proliferation protocol; however, only 13 presented data on the effect of gender on cell proliferation. Six studies found a gender effect on cell proliferation. One study showed a statistically significant increase in MSC proliferation from male donors compared to females [25]. Five studies showed a statistical difference with an increase in proliferation from female donors in comparison to male donors. Seven studies showed no statistical differences in proliferation rates between the genders [1,7,33,34,36,37,38]. Three studies failed to report the differences in proliferation rates [24,26,35]. This heterogeneity may be explained due to the variation in study methodology and characterization of cells and no clear uniformity in study protocols yielded variable results.

### 3.4. MSCs Characterization

Out of 16 studies, 14 conducted cell characterization. However, two studies did not mention how they characterized the cells [24,25]. One of these studies mentioned that the cells were commercially purchased, indicating that characterization was likely performed by the industry [25]. Two studies mentioned that they characterized the cells, but did not present any results in correlation to gender [26,38]. Seven studies did not find a correlation between gender and cell characterization [1,27,33,34,35,36,37]. High variability in MSC characterization as a result of variations in methodology was noted between the different studies. The most common implementation was flow cytometry with immunologically labeled antibodies. All studies adhered to ISCT guidelines for MSC characterization, utilizing CD90, CD73, and CD105 as positive markers, while CD45, CD31, CD34, and HLA-DR were used to exclude hematopoietic and endothelial cells. Some studies also examined CD36 as an opposite marker. Additionally, human MSCs were confirmed to express CD44 and CD166 by some of the studies [26,27,28,29,35,36].

Additionally, some studies used flowcytometry as part of the differentiation experiment to identify chondrogenic, adipogenic or chondrogenic cells (positive labeling) and markers of other cellular types (i.e., lipogenic, vascular and other unwanted cells) which were identified and removed from the analysis of data (negative labeling).

### 3.5. Chondrogenic Differentiation

Of the 16 studies identified in this literature review, seven studies specifically looked at the ability of MSCs to differentiate to the chondrogenic lineage and compared this between both males and females (Table 3) [1,27,29,34,35,36]. Although the study protocols remained heterogenous throughout with varying methods that were used to identify chondrogenic cell lineage, five of these studies did not demonstrate any statistical difference between the two sexes. One study utilized a commercial chondrogenesis differentiation kit and confirmed trilineage differentiation using Safranin O staining; however, the analysis of sex differences in chondrogenic differentiation was not presented [34]. On the other hand, Scibetta et al. (2019) found that male human muscle-derived stem cells (hMDSCs) displayed superior chondrogenesis compared to female MDSCs. While the analysis of chondrogenic markers such as Sox-9 and BMPR2 showed no detectable differences in gene expression between male and female cells, Alcian Blue staining revealed that male cells formed more robust cartilage matrices compared to female cells. Quantitative analysis confirmed a significantly higher matrix content in male hMDSC pellets for specific sample pairs. Additionally, immunohistochemistry demonstrated more intense Col2A1 staining in male hMDSC populations than in their female counterparts [35]. One study did not find differences based on sex for chondrogenic differentiation but noted that any observed variations were likely attributable to age [26]. Of the seven studies, both Scibetta et al. (2019) and Hermann et al. (2019) were the most robust of the studies as they used three different methodologies to characterize their chondrocyte-differentiated MSCs.

### 3.6. Osteοgenic Differentiation and Adipogenic Differentiation

Osteogenic differentiation was investigated in 9 of the 16 studies which reported findings (Table 4). Of the nine, five studies did not show a statistically significant difference between both male and female donors. Additionally, one study indicated a correlation between osteogenic differentiation and TNAP [1] while another study found a correlation between the site of MSCs and osteogenic differentiation [33]. One study did not analyze the difference between gender and osteogenic differentiation despite identifying these cells [34]. Three studies, however, do demonstrate a statistical significance in male donor osteogenic cells exhibiting an increased rate of proliferation and density of mineralization. Aksu et al. (2008) [24] presented gender differences in osteogenic differentiation. It started within a week, marked by vertical growth, lacunae formation, increased matrix volume, and mineralization. Notably, males exhibited faster and more efficient differentiation, particularly in the superficial depot, compared to females. Male AMSCs from both depots showed superior osteogenic potential, with those from the male superficial layer demonstrating the highest efficiency. Scibetta et al. (2019) demonstrated male human muscle-derived stem cells (hMDSCs) exhibited a greater capacity for osteogenesis compared to female MDSCs [35]. Kolliopoulos et al. (2023) revealed significant sex-based differences in mesenchymal stem cells (MSCs). MSCs from females exhibited a notably higher osteogenic response, including increased alkaline phosphatase activity, osteoprotegerin release, and mineral formation in vitro. Specifically, the study reported that female MSCs exhibited higher alkaline phosphatase (ALP) activity increased mineral deposition and elevated the secretion of SPARC, an osteogenic factor, compared to male MSCs [25]. In these studies, the authors show the superior density of matrix mineralization compared to female donors but do not appear to explain the reasons behind this.

Adipogenic differentiation was reported in five studies. Four did not demonstrate a difference between the sexes (Table 4). One study [34] did not analyze any differences between sexes despite identifying and characterizing adipogenic cells from MSCs.

### 3.7. Other Cell Differentiation Analyses

Two papers looked at the cells from muscle precursor cells: [31,32]. Of these, Riddle et al. (2018) were unable to show a statistical significance between the two groups [32]. Stolting et al. (2017) were able to show decreased sarcomeric protein expression in male donors and increased precursor-like characteristics compared to female donors [31]. One study looked at perivascular mesenchymal stem cells and demonstrated no significant differences in proliferation between the sexes [30]. In another study, researchers investigated the differentiation of angiogenic factors and the expression of genes involved in immunomodulation. Notably, they observed that angiogenic gene expression showed higher fold changes in male cells during passages 4 to 5. Conversely, in passages 3 to 5, female cells demonstrated increased expression. Additionally, male cells exhibited greater fold changes in the expression of immunomodulatory factors and genes [25].

### 3.8. Quality of Studies

A modified version of the OHAT tool was utilized to evaluate each study, applying certain criteria from the 11 questions outlined in the table below. In summary, 12 studies were determined to have a low risk overall. However, all 16 studies were identified as having a high risk in the areas of “Confidence in outcome assessment (including assessor blinding)” and likely a high risk in “Blinding of research personnel” (see Table 5). Additionally, five studies raised concerns regarding “Accounting for important confounding/modifying variables [39]”, while four studies presented issues related to “Incomplete outcome data”. Despite these specific concerns, none of the studies were categorized as high risk. All 16 studies included in this review were ultimately deemed to be of low risk and high quality.

## 4. Discussion

The role of gender in the differentiation potential of MSCs remains an area that requires further research, as it is not clear whether gender influences. Furthermore, it is not clear whether this influences the capacity of MSCs to differentiate into chondrogenic, adipogenic or osteogenic cells. Understanding this may be able to inform the researcher into the biochemical drivers behind cell differentiation that may be underpinning and driving superior lineage differentiation in the hope of optimizing conditions to transplant these cells in vivo to patients with varying osteoarthritic conditions afflicting cartilage and bone.

Of the 16 studies identified, 7 studies looked at the role of sex in the ability of cells to differentiate into chondrocytes, and 4 studies showed that sex did not demonstrate any statistical significance in their cell differentiation. This appears to imply that in both cell populations from different sexed donors, there is no influence on the ability of an MSC to drive towards a chondrogenic lineage.

On the other hand, two studies out of six were able to demonstrate that male donors preferentially drove both proliferation rates and osteogenic cell lineage from those MSCs deriving from male donors. Studies [24,35] appear to show that the abundance of mineralized matrix was statistically significant in those MSCs from male donors as opposed to female ones. Aksu et al. (2008) demonstrated that the speed of osteogenic differentiation is faster in males, whereas Scibatta et al. (2019) showed a more rapid rate in the initial first two weeks of cell induction but a plateauing rate at the four- and six-week mark where this becomes equivocal and comparable to females. West et al. (2016) concluded that these may show that male donors can commit early to in vitro myogenesis but not in vivo, where female cells appear to show more propensity for differentiation.

Three of the studies looked, additionally, at the role of age donor on the MSCs. The study found that donor age minimally impacts MSC differentiation. Adipogenesis was consistent across age groups, while chondrogenesis significantly increased in donors aged 30s and 50s by day 16. Older donors may offer enhanced potential for cartilage repair therapies [26]. The study found that age does not significantly impact the chondrogenic potential of bone marrow-derived MSCs in elderly patients (60+). MSCs from older donors, including those with osteoarthritis or fractures, effectively differentiated into chondrocytes, showing consistent therapeutic potential for cartilage repair across ages and sexes [27]. Age affects muscle progenitor cells (MPCs) differently by sex. Older male donors show impaired MPC expansion, increased cell death, and altered metabolism, while older female donors maintain their cells’ function and metabolic balance. These findings highlight a greater age-related decline in MPCs from males than females [32].

It is well established that bone density and mineralization are stronger in men in comparison to women and is related to hormonal influences on osteogenesis with the role of testosterone which increases the rate and density of bone deposition. Equally, it is believed that this hormonal influence explains the differing predisposition of post-menopausal women to low bone mass and susceptibility to fragility fracture with the reduced bone density and loss of estrogen-derived homeostasis of bone metabolism. These two studies, however, appear to show a further mechanism may be at play which has been not previously appreciated in allowing increased osteogenic differentiation in male cells. Given that these studies are performed in vitro, it would be difficult to explain this as a result of hormonal transcription factors influencing bone morphology, but they may represent pathways relevant to associated genes which are present in male cells in comparison to female cells. This remains unclear and more exploration would be required through the use of genomic studies and transcriptomics to interrogate these interplaying factors. Further analysis of these genetic pathways through genomic and transcriptomic studies may help shed light as to which genes influence these male cells compared to female cells.

The difficulty however in this systematic literature review remains the variation in the different protocols used to culture, identify and characterize adipogenic, chondrogenic and osteogenic cells. As a result, many of the results appear to show no significant difference between both sexes. Still, given the variability of the results, it is difficult to know if this is a result of the methodology utilized to culture these cells as well as tissue source, patient age and broader health status. Other confounding variables include the heterogeneity of the MSC source from various tissues as well as variations in donor age and a lack of reporting of the general health status of donor patients. Uniformity in the protocols used may provide researchers with a better understanding as to whether gender influences these cells. This remains one of the difficulties in trying to ascertain a meaningful answer on whether gender influences MSC cell differentiation.

## 5. Conclusions

This review underscores the absence of a consensus regarding the influence of gender on the chondrogenic and adipogenic potential of MSCs while suggesting a potential role in osteogenic potential. Additionally, it highlights that gender may affect cell characterization and proliferation. However, due to the diverse methodologies employed in existing studies, conclusive statements about gender’s impact on cell differentiation and proliferation remain challenging. Standardization of methodologies could facilitate future investigations into gender-specific effects on MSC differentiation and proliferation. Furthermore, the site of MSCs and age have emerged as influential factors, prompting the recommendation for a comprehensive study with uniform protocols to examine the interplay of gender, age, and site on MSC properties. Such research could inform the selection of optimal tissues for regeneration and musculoskeletal therapies.

## Figures and Tables

**Table 1:** General characteristics of the studies.

References	Brief Description of Study	Source of MSCs	Number and Gender of Subjects
Aksu et al. 2008 [24]	The research investigated the in vitro osteogenic differentiation of AMSCs, focusing on the influence of variables such as gender, fat depot location, and the ideal duration for differentiation.	-Human AMSCs -Site: superficial and deep adipose layers	Total: 6 3 females, 3 males
Garcia-Alvarez et al. 2011 [27]	The research focused on analyzing the phenotype and chondrogenic differentiation potential of bone marrow-derived MSCs obtained from elderly patients undergoing surgery for knee osteoarthritis or femoral fractures.	-Human BMSCs -Site: from femoral shaft in patients	Total: 20 12 females, 8 males
Fossett et al. 2012 [28]	This in vitro research explored the impact of age and gender on the proliferation rate and cell surface characteristics of MSCs derived from synovial fat pads. Additionally, the study examined how seeding density influences proliferation.	-Human SFPMSCs -Site: knee	Total: 14 6 females, 8 males
Kim et al. 2012 [1]	The research explored the relationship between TNAP activity in undifferentiated bone marrow MSCs and their ability to differentiate into osteocytes, chondrocytes, and adipocytes. It also analyzed the expression of osteogenic-related genes and pluripotency markers in both TNAP+ and TNAP- cells.	-Human BMSCs -Site: posterior iliac crests of iliac bone	Total: 52 29 females, 23 males
Siegel et al. 2013 [29]	Bone marrow MSCs obtained from donors were evaluated for their phenotype, growth in vitro, colony-forming ability, and telomerase activity. Functional analysis included their differentiation potential in vitro, capacity to suppress T cell proliferation, cytokine and trophic factor secretion, and the expression of receptors for hormones and growth factors. Additionally, the expression levels of Oct4, Nanog, Prdm14, and SOX2 mRNA were compared with those of pluripotent stem cells.	-Human BMSCs -Site: not specified	Total 53 25 female, 28 male
West et al. 2016 [30]	Flow cytometry analysis of 131 adipose tissue donors assessed the frequency of PSC (native ancestors of MSC) and correlated it with age, sex, BMI, and tissue cold storage time. Patient sex minimally affected the number and viability of PSC.	-Human AMSCs and adventitial cells (collectively called PSC) -Site: abdominal fat	Total: 131 112 female, 19 male
Stolting et al. 2017 [31]	Human MPCs were isolated to assess how donor age and gender influence their capacity to generate functional bioengineered muscle. The research evaluated cell yield, proliferation, and molecular expression in vitro, alongside the formation of muscle tissue and the contractile properties of the bioengineered muscle.	-Human MPCs -Site: rectus abdominis	Total: 41 18 female, 23 male
Riddle et al. 2018 [32]	The research examined the influence of donor sex and age on the expansion and metabolic preferences of human MPCs. MPCs derived from both young and older male and female donors were cultured over 17 days. Key parameters measured included confluence percentage, counts of live and dead cells, glucose consumption, and the expression of genes associated with glycolysis and oxidative phosphorylation (OXPHOS).	-Human MPCs -Site: vastus lateralis muscle	Total: 21 12 female, 9 male
Reumann et al. 2018 [33]	The study investigated the proliferation, stem cell properties, and osteogenic differentiation potential of human MSCs derived from adipose tissue collected from different subcutaneous fat sites at a Level 1 trauma center. Particular attention was given to the proliferation behavior of cells undergoing osteogenic differentiation.	-Human AMSCs -Site: abdomen, limb, hip, thigh, and knee	Total: 58 39 female,19 male
Barreto-Durán et al. 2018 [34]	The purpose of this study was to examine the effect of bone marrow donors’ clinical characteristics on the suitability of the tissue as a source of MSCs for therapeutic purposes.	-BMSCs -Site: hip	Total: 70 49 female, 21 male
Scibetta et al. 2019 [35]	This study characterized chondrogenic and osteogenic sex differences in human MDSCs using microCT, histology, and immunohistochemistry. Eight populations of MDSCs were generated as pellets in vitro and in vivo, with osteogenic potential compared between sexes.	-Human MDSCs -Site: skeletal muscle Commercially	Total: 8 4 female, 4 male
Herrmann et al. 2019 [36]	The phenotypic evaluation of BMSC cultures derived in vitro included an analysis of growth kinetics and their capacity for chondrogenic, osteogenic, and adipogenic differentiation. These characteristics were compared across various genders, donor ages, and anatomical sites.	-Human BMSCs -Site: vertebral body, iliac crest and femoral head.	Total: 33 10 female, 23 male
Lee et al. 2019 [26]	The study explored demographic influences on adipogenic and chondrogenic differentiation in BMSC spheroids. While adipogenic potential remained consistent across age groups and genders, chondrogenic potential notably increased in participants aged 30s and 50s compared to those in their 20s by day 16.	-Human BMSCs -Site: not specified	N/A
Mckinnirey et al. 2021 [37]	Demonstration of female AMSCs exhibit greater immunomodulatory potency in vitro compared to male MSCs, suggesting potential implications for donor selection in therapeutic applications.	-Human AMSCs -Site: Stomal Vascular Fraction (SVF).	Total: 8 4 female, 4 male
Lee et al. 2021 [38]	The study examined age and sex effects on viability and osteogenic potential of BMSCs cultured for bone formation. Samples from individuals in their 20s, 30s, and 50s, both male and female, were analyzed for morphology, viability, marker expression, and mineralization potential.	-Human BMSCs -Site: iliac crest	Total: 10 4 female, 6 male
Kolliopoulos et al. 2023 [25]	The study explores the effects of donor variability, passage number, and donor sex on MSC proliferation, osteogenic capability, and regenerative potential within mineralized collagen scaffolds. Findings highlight notable variations influenced by donor sex and the length of passage.	-Human BMSCs -Cadaveric AMSCs. -Site: not specified	Total: 8 4 female, 4 male

AMSCs = Adipose-derived Mesenchymal Stem Cells; TNAP = Tissue Nonspecific Alkaline Phosphatase; MPC = Muscle Precursor Cell; MDSC = Muscle-Derived Stem Cells; BMSC = Bone Marrow-derived Stem Cells; SFPMSCs = Synovial fat pad mesenchymal stem cells; N/A= Not Applicable.

**Table 2:** Proliferation and MSC characterization.

References	Proliferation Protocol	Proliferation Results	MSCs Characterization	Other Outcomes
Aksu et al. 2008 [24]	AMSCs were maintained in DMEM/F-12 medium enriched with 10% heat-inactivated FBS, 1% penicillin/streptomycin, and 1% Fungizone (Gibco, Invitrogen Corporation). The cells were cultured at 37 °C in a 5% CO_2_ environment for 5–8 days, with the medium replaced every two days until reaching 80% confluence. Cells at passage 1 were utilized for the experiments.	N/A	N/A	N/A
Garcia-Alvarez et al. 2011 [27]	Nucleated cells were plated at 10^4^ cells/cm^2^ in b-FGF supplemented medium. They reached 80–90% confluence by day 14. Subsequently, cells were replated at 2000 cells/cm^2^ and cultured for 7 days with medium changes twice weekly for proliferation analysis.	Proliferation data showed no difference associated with gender.	No statistical differences between gender in flow cytometry when looking at passage 1 BMSC expression of CD13, CD34, CD44, CD105, CD166, CD31, CD36, CD45, CD49c, CD49d, CD49f, CD59, CD71, CD73, CD90, CD106, CD117, CD151, CD271, CD133/1, CD54, HLA-DR. CD45, CD34 and CD31 used as negative markers. CD36 used as adipocyte markers.	CD marker analysis revealed no significant differences between donors. MSCs from knee arthroplasty and femoral fracture groups exhibited similar profiles, except for higher CD49d % in fracture MSCs and higher CD49f % in knee OA MSCs at passage 1. MSCs from elderly knee OA patients could differentiate into chondrocytic lineages with no discernible variations compared to MSCs from femoral fracture patients.
Fossett & Khan et al. 2012 [28]	Passage 2 cells were plated at densities ranging from 50 to 10,000 cells/cm^2^. The proliferation rate was evaluated using Alamar Blue assays conducted on days 0, 3, 6, 10, 14, 17, and 21. Population doublings per day were determined using the formula: ln(N/N_0_)/t, where N represents the cell count on the specified day, N_0_ is the cell count at day 0, and t is the time in days.	Female-derived MSCs (n = 5) exhibited a higher rate of population doublings per day compared to male-derived MSCs (n = 7) across all seeding densities. However, these differences were not statistically significant.	Cell surface staining and flow cytometry were conducted to analyze markers including CD34, CD44, CD80, CD90, CD45, CD54, CD73, CD106, CD166, STRO-1, and CD105. The mean expression of all markers was higher in females (n = 4) compared to males (n = 7). A statistically significant difference was observed for STRO-1 expression (*p *= 0.039), while no significant differences were found for the other markers. CD105 consistently maintained stable expression levels.	An inverse correlation between seeding densities and cell proliferation rates was observed, with the relationship being statistically significant.
Kim et al. (2012) [1]	BMSCs at passage 1 were seeded at a clonal density of 1 × 10^2^ cells per 10 cm^2^ culture dish. Over time, these cells formed colonies, which were isolated using cloning cylinders placed over the colony-forming cells. Individual clone-derived colonies were carefully detached and cultured in growth medium for 12 days. Proliferation of the cells was assessed using the MTT assay.	In proliferation assays, TNAP+ sorted cells demonstrated lower proliferation rates compared to TNAP- sorted cells. Real-time quantitative PCR revealed that the expression of cell cycle-related genes, including cyclin A2, CDK2, and CDK4, was higher in TNAP- bone marrow MSCs. Additionally, TNAP expression levels showed no association with gender.	Cells were analyzed using flow cytometry, and no differences were observed in the expression of CD105 and CD29 between TNAP+ and TNAP- donors, with expression levels at 76% and 77%, respectively.	TNAP+ cells naturally express several transcription factors related to osteogenesis, such as cbfa1 and dlx5, with their expression levels increasing over time in culture. Initial TNAP activity in unexpanded BMSCs displayed variability among donors but showed no correlation with donor age or gender.
Siegel et al. 2013 [29]	To evaluate colony formation capacity, passage 1 BMSCs were plated at densities of 100, 200, and 500 cells per well in six-well plates for 10 days. The percentage of colony formation, calculated as the number of colonies formed per 100 seeded cells, was determined for each seeding density in each MSC preparation.	At a density of 1000 cells per cm^2^ at P1, faster-dividing cells were found to consist of subpopulations with higher expression levels of surface markers. While no significant correlation was observed between donor gender and proliferation capacity (n = 52), MSCs with high clonogenic potential were smaller, divided more quickly, and were more commonly found in samples from younger female donors. Gender differences also affected cell diameter, with BM-MSCs from female donors (20.9 μm ± 0.8 μm SD) (n = 14) being slightly but significantly smaller compared to those from male donors (22.0 μm ± 1.1 μm SD).	MSC preparations derived from female donors contained a significantly higher number of CD119+ cells (n = 11) and CD130+ cells (n = 16) when compared to those from male donors (CD119+ cells: n = 17; CD130+ cells: n = 19). Additionally, MSCs obtained from younger donors exhibited elevated expression levels of MCAM, VCAM-1, ALCAM, PDGFRβ, PDL-1, Thy1, and CD71. Conversely, the expression of CD71, CD90, CD106, CD140b, CD146, CD166, and CD274 demonstrated a negative correlation with donor age.	Bone marrow MSCs exhibited significantly lower mRNA expression levels of Oct4, Nanog, and Prdm14 compared to pluripotent stem cells, with no observed correlation to donor age or gender. The clonogenic potential of MSCs showed a positive association with Prdm14 mRNA expression (n = 18), while no such correlation was found with Oct4 (n = 24) or Nanog mRNA (n = 24).
West et al. 2016 [30]	The lipoaspirate was processed by washing and centrifugation, followed by digestion with type II collagenase for 45 minutes. After digestion, the samples were centrifuged to isolate the stromal vascular fraction (SVF), which was then filtered. Live cells were manually counted using trypan blue staining and a hemocytometer.	The average number of SVF cells obtained from 100 ml of lipoaspirate was 34.4 million. No significant differences were found in SVF cell yield (*p* = 0.34) or viable PSC counts (*p* = 0.79) between male and female donors. However, the proportion of PSCs among live cells was significantly higher in male donors, with mean proportions of 47% compared to 40% in females (*p* = 0.05, one-tailed t-test). Despite this, the average SVF yield (males: 30 × 10⁶ cells vs. females: 35 × 10⁶ cells) and cell viabilities (males: 82% vs. females: 83%) were slightly lower in males, though not significantly, leading to an overall balance in outcomes. The number and viability of these cells showed minimal variation between genders.	The SVF was analyzed using staining for CD31, CD34, CD45, and CD146. CD45+ cells accounted for an average of 34% (SD: ±16%; n = 113), while CD31+ cells comprised 4% (SD: ±4%; n = 17) of the total live cells in the SVF. The mean proportion of PSCs was 41% (SD: ±16%; n = 124), with pericytes and adventitial cells making up 8% (SD: ±8%; n = 131) and 33% (SD: ±16%; n = 131), respectively. This equates to an average yield of 11.6 × 10⁶ PSCs (SD: 8.6 × 10⁶; n = 124) per 100 ml of lipoaspirate. The number and viability of these cells remain largely unaffected.	Adipose tissue was stored at 4 °C for up to 7 days before analysis. When evaluated at specific time intervals using the Tukey–Kramer (honest significant difference) test, a general, though not statistically significant, increase in the proportion of PSCs within the SVF was observed over time. This relative rise was attributed to a decline in the proportion of CD45+ hematopoietic cells, as the absolute number of PSCs remained stable.
Stolting et al. 2017 [31]	Growth curves were generated by seeding MPCs at a density of 5000 cells/cm^2^ in 24-well plates using passage 1 cells. Total cell counts were recorded over time and plotted to reflect growth trends. Cell cycle rates were derived from the average doubling times at the midpoint of the growth curve for each cell type. Cell proliferation was monitored daily for one week, and viability was evaluated using toluidine blue staining, with live cells expressed as a percentage.	Cells derived from female donors exhibited greater growth and yield compared to those from male donors. By day 4, the number of cells in P1 from female samples was nearly double that of male samples (female: 44.06 ± 0.44 × 10⁶ cells/cm^2^; male: 2.43 ± 0.24 × 10⁶ cells/cm^2^; *p* < 0.05). Similarly, the precursor cell yield per gram of muscle tissue was approximately twice as high in female samples (female: 11.07 ± 1.31 × 10⁵ cells/g; male: 4.02 ± 0.38 × 10⁵ cells/g; *p* < 0.001).	Primary antibodies used included anti-desmin, anti-myosin heavy chain, anti-MyH1, anti-MyoD, anti-Pax7, anti-PGP 9.5, and monoclonal anti-GAPDH. No differences were observed in Pax7 or desmin expression between male and female groups. However, male cells exhibited significantly higher expression of MyoD and sarcomeric markers, such as sarcomeric α-actinin and MyHC (*p* < 0.001, *p* < 0.05, *p* < 0.05, respectively). Additionally, a greater proportion of male cells expressed both myosin types, while female cells predominantly remained undifferentiated at P3.	Cells from female donors exhibit improved innervation and greater strength, with a preference for forming slow-twitch muscle fibers following transplantation.
Riddle et al. 2018 [32]	MPCs were plated at approximately 10% density in 96-well plates and allowed to proliferate over 17 days. Measurements of live cell count, percentage of dead cells, and culture confluence were recorded every 24 h.	YF-MPCs (young female) displayed a higher percentage of dead cells, fewer live cells, and lower confluence and saturation density compared to YM-MPCs (young male). However, no differences in population doubling times were observed among young hMPCs. OM-MPCs (old male) exhibited a higher percentage of dead cells and longer population doubling times compared to OF-MPCs (old female). In contrast, no significant differences were found in the total percentage of dead cells, live nuclei, confluence, or saturation density within the old hMPC group. The authors concluded that aging has a pronounced impact on male hMPCs, with OM-hMPCs showing reduced expansion capacity compared to YM-hMPCs.	Approximately 1–1.5 million cells were stained with fluorescently conjugated antibodies targeting myoblast surface markers CD56 (NCAM) and CD29 (beta1-integrin), along with the viability dye 7-aminoactinomycin D. Sorting resulted in 150,000–800,000 viable CD56/CD29 MPCs per million cells. The sorted cells, which were 98% positive for CD56/CD29, were expanded in culture through two passages before being used for experiments. All in vitro studies were conducted with cells at passage 6. No gender-based comparisons were made regarding cell surface marker expression.	The metabolic properties of MPC cultures during expansion were assessed using Seahorse flux analysis. The metabolic phenotype was evaluated by measuring the extracellular acidification rate (ECAR), as an indicator of glycolysis, and the oxygen consumption rate (OCR), as an indicator of oxidative phosphorylation (OXPHOS). YF-MPCs exhibited a significantly lower baseline OCR compared to YM-MPCs, though no significant differences were observed in OCR metabolic potential. In contrast, OF-MPCs showed no significant difference in baseline OCR compared to OM-MPCs but demonstrated a significantly reduced OCR metabolic potential. No significant main effects or interactions were identified for any ECAR measurements.
Reumann et al. 2018 [33]	Passage 3 cells were plated at 10^4^ cells/cm^2^ and their proliferation was assessed	SRB staining showed no difference in amounts of cells immediately after plating. Within 14 days there was a significant increase in MSCs derived from hip and thigh (10% and 6% increase, respectively). Increases of 61% and 65% were detected in knee and abdomen, respectively. MSCs of the limb showed the biggest growth at 81%. The proliferation potential of MSCs showed no significant variation based on donor gender.	At passage 3, flow cytometry analysis demonstrated that MSCs were positive for CD73, CD90, and CD105, while showing negative expression for CD14, CD45, and HLA-DR. No comparisons between genders were made for cell surface marker.	N/A
Barreto-Durán et al. 2018 [34]	Mononuclear cells derived from bone marrow were isolated using Ficoll density gradient centrifugation and subsequently plated. The time to confluence was defined as the duration required to achieve fibroblastoid adherent cells with over 70% confluence.	No notable associations were found between donor gender and the expansion properties of hMSCs. Similarly, no significant correlation was detected between donor age and the volume of bone marrow collected or the total number of isolated mononuclear cells. However, a significant relationship was identified between donor age and the time required for cells to reach confluence in vitro (r = 0.2489, *p* = 0.0377).	The phenotype of BMSCs was characterized by analyzing markers CD73, CD105, CD45, and CD34. The MSCs were negative for hematopoietic markers CD34 and CD45, while showing co-expression of CD73 and CD105.	N/A
Scibetta et al. 2019 [35]	The hMDSCs were grown and maintained in high-glucose DMEM with 20% fetal bovine serum, 1% chicken embryo extract (100–163P; Gemini Bio-Products, West Sacramento, CA, USA), and 1% penicillin/streptomycin.	N/A	CD105, CD73, CD90, CD56, and CD44 were used to characterize the MDSCs. MDSCs express CD73, CD90, CD104, and uniquely express CD56. All pairs of male and female No gender difference for CD44, CD56, CD73, CD90, and CD105.	N/A
Herrmann et al. 2019 [36]	BMSCs were seeded and population doubling time was measured	Bone marrow source and gender did not affect cell yield or population doubling time.	After selection and expansion on tissue culture plastic, BMSC lines exhibited a typical cell surface marker profile, characterized by positive expression of CD44, CD90, CD73, and CD105. CD146 expression was consistently high in cells from the iliac crest and vertebral body, whereas MSCs derived from the femoral head displayed more variable expression levels at p0 and p1. No gender related differences were noted.	A trend was observed between the abundance of CD146+NG2+ cells and population doubling time, though it did not reach statistical significance. Similarly, a negative correlation trend was noted for the abundance of CD45+ leukocytes, but this too was not statistically significant.
Lee et al. 2019 [26]	Human BMSCs were plated in 24-well plates at a density of 2 × 10⁴ cells per well and cultured at 37 °C in a medium containing 15% FBS, 100 U/ml penicillin, 100 µg/ml streptomycin, 200 mM L-glutamine, and 10 mM ascorbic acid 2-phosphate.	N/A	Tests by the Catholic Institute of Cell Therapy confirmed high CD73 and CD90 expression (>90% positive).	N/A
Mckinnirey et al. 2021 [37]	AMSCs were isolated, cultured from thawed SVF, and expanded to ~1 × 10⁸ cells per donor after four passages. The MSC secretome (MSC-S) was collected, centrifuged, and stored at −80 °C. MSCs were stored at 2 × 10⁶ cells per aliquot in CS10 and transferred to liquid nitrogen. Cell count and viability were assessed using flow cytometry with Propidium Iodide and SYTO 11 staining.	Male and female MSCs displayed comparable growth rates and phenotypic traits. No significant differences were observed in MSC doubling per day or total doublings between donors up to passage 4, and all MSCs exhibited a fibroblast-like morphology.	AMSCs from age-matched male and female donors were cultured to passage 4 and analyzed for population doubling, protein expression, and immunophenotyping. MSCs were exposed to inflammatory cytokines and PBMCs from male and female donors for further assays. Flow cytometry confirmed that both male and female MSCs displayed typical MSC markers, being negative for CD45, CD34, CD11b, HLA-DR, and CD19, and positive for CD90, CD105, and CD73. Extended analysis showed consistent MSC characteristics across all samples. PBMC proliferation assays revealed that both male and female MSCs effectively suppressed PBMC proliferation, with immune suppression rates reported for all groups.	Female adipose-derived MSCs (fMSCs) exhibit stronger immunomodulatory effects than male MSCs (mMSCs). fMSCs more effectively suppressed PBMC proliferation and sustained higher levels of VCAM-1 after inflammation, while mMSCs maintained HLA-DR expression. fMSCs also produced higher levels of immunosuppressive factors like IDO1, PGE-2, and IL-1RA. Inhibiting IDO1 significantly reduced fMSCs’ suppressive ability, highlighting its importance. These findings suggest fMSCs may be more effective in MSC therapy due to their superior immunosuppressive properties.
Lee et al. 2021 [38]	BMSCs at passage 2, were used and 1.0 × 10⁶ BMSCs were plated at different densities, and grown in an osteogenic medium. Cell morphology was evaluated on Days 1 and 3, with differences analyzed across age groups (20s, 30s, and 50s). Viability was assessed using a LIVE/DEAD Kit on Day 4 and a WST-8 assay on Days 1 and 3, with absorbance measured at 450 nm. Experiments were repeated three times.	On Day 1, BMSCs displayed fibroblast-like shapes, with fewer cells observed in the 50s and 30s groups, particularly in 30s females. Viability testing using a LIVE/DEAD Kit showed most cells were alive, but more dead cells were found in the 20s female, 30s male, and 50s female groups. The CCK-8 assay revealed no significant differences in viability among age groups on Days 1 and 3. However, there were significant differences in viability between male and female groups on both days, with females showing lower viability (*p* < 0.05).	The Catholic Institute of Cell Therapy confirmed >90% positivity for CD73 and CD90, while CD31, CD34, and CD45 were >90% negative. Results not mentined.	N/A
Kolliopoulos et al. 2023 [25]	Passage 3 human MSCs from four donors were cultured in T175 flasks with RoosterNourishTM-MSC at 37 °C and 5% CO_2_, expanding to p3-5 or p3-6, while Passage 4 MSCs expanded to p4-5. Cells were seeded at 150,000 cells/scaffold onto mineralized collagen, cultured for 21 days in supplemented DMEM, with media changes every three days and aliquots stored for ELISA.	Male donors generally show higher metabolic activity and increased cell proliferation rates compared to female donors.	Not mentioned. Commercially bought indicating that characterization was done industrially.	N/A

b-FGF = fibroblastic growth factor-basic; SVF = stromal vascular fraction; SRB = sulforhodamine B; BMSC = bone marrow-derived stem cells; AMSCs = adipose-derived mesenchymal stem cells.

**Table 3:** Chondrogenic and Osteogenic Differentiation.

Reference	Chondrogenic Protocol	Chondrogenic Results	Osteogenic Protocol	Osteogenic Results
Aksu et al. 2008 [24]	N/A	N/A	Osteogenic differentiation was evaluated over 1, 2, and 4 weeks using osteogenic medium, with cells seeded at a density of 50 × 10^3^ cells per well. ALP, alizarin red, and Masson’s trichrome staining were used to assess osteoblastic differentiation, matrix mineralization, and collagen synthesis. Gla-osteocalcin levels were quantified via ELISA, while osteonectin protein expression was analyzed through Western blotting.	Osteogenic differentiation began at 1 week, marked by vertical growth, lacunae formation, increased matrix volume, and mineralization. Initially, prominent differences were observed in alkaline phosphatase staining. Androgen receptor activity increased progressively over 4 weeks, with collagen secretion detected only in differentiated AMSCs. Males exhibited earlier differentiation than females, supported by osteonectin and osteocalcin profiling. Male AMSCs from both depots (represent different locations of adipose/fat tissue from which the AMSCs were isolated) showed superior differentiation, especially in superficial depots. Conversely, no significant difference was found in female AMSC differentiation between depots. AMSCs obtained from male superficial abdominal adipose tissue demonstrated the highest efficiency in achieving osteogenesis.
Garcia-Alvarez et al. 2011 [27]	Micromasses were formed by pelleting 200,000 cells in a 15-mL tube and then cultured in chondrogenic medium for 30 days. The medium contained TGF-β3, dexamethasone, ascorbate-2-phosphate, proline, ITS + Premix, and BMP-6.	Safranin-O staining was conducted to assess proteoglycans and glycosaminoglycans. Samples were graded based on a histological scale evaluating uniformity and darkness of stain, matrix accumulation, and cellular morphology. No differences were observed between male and female patients in terms of histological differentiation grading or cellular marker expression.	N/A	N/A
Fossett et al. 2012 [28]	N/A	N/A	N/A	N/A
Kim et al. 2012 [1]	A 10 mL volume of suspended cells, at a concentration of 8000 cells/mL, was placed in the center of each well in 24-well plates for micromass culture. Cells were then cultured for 14 days using a chondrogenic medium containing DMEM-high glucose, insulin-transferrin-selenium-A, 50 µg/mL ascorbic acid, and 10 ng/mL TGF-β3.	The study revealed higher levels of type II collagen in TNAP- cells compared to TNAP+ cells. Conversely, TNAP+ BMSCs exhibited increased expression of type X collagen and cbfa1, associated with hypertrophic tissue and osteoarthritic cartilage. Safranin-O staining indicated greaterproteoglycan synthesis in TNAP- cells compared to TNAP+ cells.	Bone marrow MSCs from passages 1 to 3 were maintained in an osteogenic medium consisting of DMEM supplemented with 10% FBS, 0.1 mM dexamethasone, 50 µg/mL ascorbic acid, and 10 mM β-glycerophosphate for a duration of 14 days.	Osteogenic gene expression (cbfa1, dlx5, osterix, dlx5, and osteopontin) and Von Kossa staining was performed. During osteogenic differentiation, both cell types (TNAP-, TNAP+) displayed comparable mRNA expression profiles on day 5, with a reduction in msx2 and osterix expression levels, while *dlx5* and *cbfa1* increased. Calcium content indicated a positive correlation between *TNAP* level and osteogenic differentiation ability.
Siegel et al. 2013 [29]	Differentiation was initiated using the commercially available MSC Chondrogenic Differentiation BulletKit (PT-3003, Lonza), with the addition of TGF-β3 as a supplement.	No significant statistical differences were observed in the lineage-specific mRNA expression levels of SOX9 (n = 47) and COLL2 (n = 32), as well as in the Safranin O staining results (n = 40).	Osteogenic differentiation was triggered by culturing cells in an osteogenic medium supplemented with dexamethasone, ascorbic acid and β-glycerolphosphate.	No differences related to donor age or gender were detected in the osteogenic differentiation capacity in vitro, as assessed by lineage-specific Alizarin Red staining (n = 40). Similarly, no statistically significant variations were observed in the lineage-specific mRNA expression levels of OPN (n = 17) and ALP (n = 41).
West et al. 2016 [30]	N/A	N/A	N/A	N/A
Stolting et al. 2017 [31]	N/A	N/A	N/A	N/A
Riddle et al. 2018 [32]	N/A	N/A	N/A	N/A
Reumann et al. 2018 [33]	N/A	N/A	For osteogenic differentiation, the study employed several assessment methods on Passage 3 cells. Cells were seeded at a density of 10⁴ cells/cm^2^ and cultured for 14 days in an osteogenic medium enriched with 200 µM L-ascorbic acid 2-phosphate, 10 mM β-glycerophosphate, 25 mM HEPES, 1.5 mM calcium chloride, and 5 µM cholecalciferol. ALP Activity: This was measured to evaluate early osteogenic differentiation, with cells incubated with a substrate to quantify the resulting product. Matrix Mineralization: Mineral deposition, a key indicator of osteogenesis, was assessed using Von Kossa and Alizarin Red staining. The mineralized matrix was visualized and quantified photometrically. Sulforhodamine B (SRB) Staining: This method quantified cell numbers by measuring protein content, indirectly reflecting the extent of osteogenic differentiation. Semi-Quantitative RT-PCR: Gene expression relevant to osteogenesis (e.g., Runx2, SP7) was analyzed, providing insights into molecular changes during differentiation. Western Blot: Protein levels of key osteogenic markers were evaluated, confirming the differentiation at the protein level.	MSCs from different adipose tissue sites exhibit varied proliferation and osteogenic differentiation capacities. MSCs isolated from the hip and thigh regions exhibited the greatest osteogenic potential, characterized by increased expression of Oct4α, Lin28A, and Nanog, alongside decreased Sox2 levels. Conversely, MSCs derived from the abdominal area demonstrated robust proliferative capacity and osteogenic features, while those from the knee and limb had the strongest proliferation but limited osteogenic differentiation. The donor site significantly influenced these characteristics more than the donor’s age, weight, or gender. Therefore, selecting the adipose tissue site should be based on whether the priority is cell quantity or osteogenic differentiation.
Barreto-Durán et al. 2018 [34]	A commercial chondrogenesis differentiation kit was used	Safranin O staining was performed to confirm trilineage differentiation and gender differences were not analyzed.	A commercial osteogenic differentiation kit was used	Red alizarin staining was performed to confirm trilineage differentiation and gender differences were not analyzed.
Scibetta et al. 2019 [35]	Three-dimensional pellets of male and female MDSCs were maintained in a commercially available chondrogenic medium for a duration of 24 days.	Both male and female MDSC pairs exhibited expression of SOX9 and BMPR2, with no notable differences observed. In vitro microCT analysis revealed comparable chondrogenic potential between male and female pellets. While Alcian blue chondrogenic staining did not reveal significant differences between grouped male and female MDSCs, male MDSCs displayed a trend towards greater chondrogenicity. Male MDSC populations exhibited stronger *Col2A1* staining compared to females.	Osteogenic differentiation media was used for 14 day (media was not mentioned). In vivo, cells were transplanted into ICR-SCID mice using LMP2/GFP virus. Herovici’s staining identified collagen type I. Hematoxylin and eosin (H&E) staining demonstrated the formation of newly developed bone and bone marrow.	Von Kossa staining indicated enhanced mineral deposition in pellets derived from male MDSCs, with calcium deposition more pronounced in males, albeit prone to peeling. Osteocalcin staining revealed no gender difference. Both male and female MDSCs demonstrated expression of RUNX2 and 15-PGDH without significant differences. *COX-2* expression varied among pairs with no clear gender discrepancy. *WNT3A* expression did not differ by gender. Male MDSC-LBMP2/GFP cells generated more bone after 2 weeks, and at 4 and 6 weeks, male LBMP2/GFP-transduced MDSCs displayed significantly greater bone density compared to their female counterparts.
Herrmann et al. 2019 [36]	Cryopreserved p2 BMSCs were cultured in chondrogenic media with TGF-β-1 and other supplements. A total of 2.5 × 10⁵ MSCs were centrifuged into pellets, which were cultured for 21 days, with the medium being refreshed every 3 days. Pellets were then fixed, cryosectioned, and stained for sulphated glycosaminoglycans (sGAG) and collagen II and X. Differentiation was evaluated based on pellet size and Safranin-O staining intensity.	A semi-quantitative histological assessment was conducted, considering pellet size and Safranin-O staining intensity. Additionally, immunostaining for collagen type II and collagen type X was carried out. All BMSC sources produced structurally stable chondrogenic pellets.	Cells were plated at a density of 20,000 cells/cm^2^ in 24-well tissue culture plates and cultured in osteogenic differentiation medium containing 50 µg/mL ascorbic acid, 5 mM β-glycerophosphate, and 10 nM dexamethasone for 21 days, with the medium replaced three times per week.	Alizarin red staining was used to evaluate mineral deposition. The results indicated that neither age nor gender had a notable impact on the abundance or multi-lineage differentiation capacity of BMSCs.
Lee et al. 2019 [26]	BMSCs were induced to undergo chondrogenic differentiation, employing a StemPro^®^ Chondrogenesis Differentiation Kit was utilized. Cells were stained with Alcian blue solution on days 8 and 16. Chondrogenesis was evaluated. The intensity of Alcian blue staining was analyzed using ImageJ software (version 1.8.0).	This study revealed no notable differences in the chondrogenic differentiation potential of BMSCs derived from healthy male and female donors. However, BMSCs from individuals in their 30s and 50s exhibited significantly greater chondrogenic differentiation potential by day 16 compared to those from individuals in their 20s.	N/A	N/A
Mckinnirey et al. 2021 [37]	N/A	N/A	N/A	N/A
Lee et al. 2021 [38]	N/A	N/A	Cells were maintained in osteogenic medium supplemented with glycerophosphate, dexamethasone, L-ascorbic acid 2-phosphate, L-glutamine, and fetal bovine serum. ALP activity was evaluated with a commercial assay kit, measuring absorbance at 405 nm. Alizarin Red S staining was conducted on days 8 and 16.	The findings indicate no notable differences in Runx2 or collagen I expression levels across different ages or genders. Likewise, alkaline phosphatase activity and Alizarin Red S staining showed no variations related to age or gender.
Kolliopoulos et al. 2023 [25]	N/A	N/A	*ALP* activity was assessed using an assay kit (Abcam, Cambridge, UK) for osteogenic differentiation on samples collected from cell-seeded scaffolds and compared against unseeded controls. Media collected every three days were grouped for analysis into five categories: Day 3, Days 6–9, Days 10–15, Days 16–30, and Days 33–56.	The passage protocol significantly influenced osteogenic functional assays. Male donors exhibited greater expression of osteogenic genes and showed increased secretion of osteogenic and immunomodulatory factors by day 56. In contrast, female donors secreted higher levels of SPARC by day 56 and demonstrated enhanced ALP activity, OPG secretion, and mineral deposition.

AMSCs = Adipose-derived Mesenchymal Stem Cells; BMP-6 = bone morphogenetic protein-6; SRB = Sulforhodamine B; hMDSCs = human Muscle-Derived Stem Cells; OPN = Osteopontin; ALP = Alkaline phosphatase; TNAP = tissue nonspecific alkaline phosphatase activity; BMSCs = Bone Marrow Mesenchymal Stem Cells; OPG = osteoprotegerin.

**Table 4:** Adipogenic differentiation and other results.

References	Adipogenic Protocol	Adipogenic Results	Other Results
Aksu et al. 2008 [24]	N/A	N/A	N/A
Garcia-Alvarez et al. 2011 [27]	N/A	N/A	N/A
Fossett & Khan et al. 2012 [28]	N/A	N/A	N/A
Kim et al. 2012 [1]	Cells were maintained in adipogenic medium, consisting of DMEM supplemented with 10% FBS, 1 mM dexamethasone, 0.5 mM IBMX, 200 mM indomethacin, and 5 mM insulin, for a duration of 14 days.	Adipogenic differentiation capacity, as assessed by Oil Red O staining, appeared to be independent of TNAP activity.	TNAP is involved in osteogenic and adipogenic differentiation of BMSCs, with no apparent role in chondrogenic differentiation. BMSCs with TNAP+ exhibited bipotential differentiation toward osteogenic and adipogenic lineages, whereas TNAP- BMSCs demonstrated multipotential differentiation capabilities, including osteogenic, adipogenic, and chondrogenic pathways. TNAP expression level showed no correlation with gender.
Siegel et al. 2013 [29]	Adipogenic differentiation using a commercial adipogenic kit	Adipogenic differentiation capacity of MSC preparations, evaluated through Oil Red O staining, showed no discernible differences related to gender or age. Similarly, no statistically significant variations were observed in the mRNA expression levels of LPL (n = 44) and PPARγ (n = 48).	N/A
West et al. 2016 [30]	N/A	N/A	N/A
Stolting et al. 2017 [31]	N/A	N/A	Samples were analyzed at 2 and 4 weeks post-transplantation for myogenic differentiation. Western blot assessed anti-MyoD, anti-Pax7, and anti-PGP, while immunolabeling included markers like anti-desmin, anti-myosin heavy chain, and anti-Pax7. Cytoskeleton staining used phalloidin, myofibers with Giemsa, and neuromuscular junctions with fluorescein and α-bungarotoxin.Results showed gender differences in MPC fate post-transplantation. Male cells had increasing expression of early myogenic markers (Pax7 and MyoD) but decreased sarcomeric protein expression over time. Female cells maintained stable Pax7 and MyHC levels, with increased MyoD and MyH1, indicating a commitment to type 1 muscle fibers. Immunohistochemistry demonstrated a progressive increase in neuromuscular junctions and innervation in both genders over time, with female samples displaying enhanced clustering of acetylcholine receptors and a greater density of nerve filaments. Protein expression analysis confirmed superior nerve ingrowth in female samples at 4 weeks. Additionally, female-derived tissues demonstrated increasing contraction force (*p* < 0.001), while male-derived tissues showed a decrease (*p* < 0.001) upon electrical tetanic stimulation (80 V, 80 Hz).
Riddle et al. 2018 [32]	N/A	N/A	N/A
Reumann et al. 2018 [33]	N/A	N/A	N/A
Barreto-Durán et al. 2018 [34]	A commercial adipogenic differentiation kit was used	Sudan black staining was performed to confirm trilineage differentiation and gender differences were not analyzed.	N/A
Scibetta et al. 2019 [35]	N/A	N/A	Herovici’s staining showed new bone formation in both male and female groups, with higher collagen density in males. H&E hematoxylin and eosin and TRAP staining confirmed functional bone formation and no differences in osteoclast activity.
Herrmann et al. 2019 [36]	Cells were plated at a density of 16,000 cells/cm^2^ in 24-well plates and maintained in adipogenic differentiation medium containing 5 µg/mL insulin, 1 µM dexamethasone, 0.5 mM isobutylmethylxanthine, and 60 µM indomethacin. The cultures were sustained for two weeks, with the medium being refreshed three times per week.	Oil Red O staining was performed, revealing that neither age nor gender had a notable impact on the quantity or multi-lineage differentiation capacity of BMSCs.	The current profiling of cell-surface markers for distinct progenitor populations in bone marrow does not effectively forecast the properties of in vitro BMSC cultures. For cartilage repair approaches, stromal progenitor cells are more reliably obtained from the iliac crest and vertebral body compared to the femoral head.
Lee et al. 2019 [26]	A BMSCs were differentiated using StemPro^®^ Adipogenesis Differentiation kit.	Adipogenesis was assessed by analyzing the intensity of Oil Red O staining on days 8 and 16 using ImageJ software. Flow cytometry was performed on days 8 and 16 utilizing a fluorescein isothiocyanate-labeled CD44 antibody. No notable differences in adipogenic differentiation potential were observed between male and female donors in good health.	N/A
Mckinnirey et al. 2021 [37]	N/A	N/A	N/A
Lee et al. 2021 [38]	N/A	N/A	Examinations of cell morphology, viability, SSEA-4 expression, and VEGF secretion revealed no substantial variation in SSEA-4 expression across different age groups or between genders. Similarly, VEGF secretion levels showed no significant differences between males and females.
Kolliopoulos et al. 2023 [25]	N/A	N/A	Male donors exhibited notably higher immunomodulatory gene expression than female donors throughout the culture period, irrespective of the passage scheme. Conversely, angiogenic gene expression varied based on the passage scheme: male cells displayed elevated levels in the p4-5 scheme, while female cells showed increased expression in the p3-5 scheme. Interestingly, VEGFA was the only angiogenic gene to demonstrate a significant shift, with higher expression in female cells under the p3-5 scheme and in male cells under the p4-5 scheme. These findings highlight the significant influence of the passage scheme on sex-specific differences in gene expression, particularly for angiogenic genes.

BMSCs = Bone Marrow-Derived Mesenchymal Stem Cells; ELISA = enzyme-linked immunosorbent assay, MPC = Muscle Precursor Cell, TNAP = tissue nonspecific alkaline phosphatase activity, H&E = hematoxylin and eosin.

**Table 5:** Risk of bias analysis/assessment.

	Clinical Trial ID															
	1	2	3	4	5	6	7	8	9	10	11	12	13	14	15	16
Randomization of administered dose or exposure level	N/A	N/A	N/A	N/A	N/A	N/A	N/A	N/A	N/A	N/A	N/A	N/A	N/A	N/A	N/A	N/A
Allocation concealment	N/A	N/A	N/A	N/A	N/A	N/A	N/A	N/A	N/A	N/A	N/A	N/A	N/A	N/A	N/A	N/A
Appropriate participant selection for comparison	++	+	++	+	++	+	++	++	+	+	++	+	+	++	++	++
Accounting for important confounding/modifying variables	-	-	-	++	+	+	+	+	+	-	+	-	+	+	+	+
Identical experimental conditions across study groups	++	++	++	++	++	++	++	++	++	++	++	++	++	++	++	++
Blinding of research personnel	-	-	-	-	-	-	-	-	-	-	-	-	-	-	-	-
Incomplete outcome data	+	+	++	++	++	-	+	+	+	-	-	+	++	+	-	+
Confidence in exposure characterization	++	++	++	++	++	++	++	++	++	++	++	++	++	++	++	++
Confidence in outcome assessment (incl. assessor blinding)	--	--	--	--	--	--	--	--	--	--	--	--	--	--	--	--
Complete reporting of measured outcomes	++	+	++	++	++	+	++	++	+	+	++	+	++	++	+	+
Other potential threats to internal validity (bias)	++	+	+	++	++	+	++	++	+	+	++	++	++	++	++	++

Trial ID: 1 = Asku (2008); 2 = Garcia Alvarez (2011); 3 = Fosset and Khan (2012); 4 = Hee Kim (2012); 5 = Siegel (2013); 6 = West (2016); 7 = Stolting (2017); 8 = Riddle (2018); 9 = Reumann (2018); 10 = Duran (2018); 11 = Scibetta (2019); 12 = Herrmann (2019); 13 = Lee (2019); 14 = Mckinnirey (2021); 15 = Lee (2021); 16 = Kolliopoulos (2023). ++ Definitely low risk; + Probably low risk; - Probably high risk; -- Definitely high risk.

## Data Availability

Not applicable.

## References

[1] Kim Y.H., Yoon D.S., Kim H.O., Lee J.W. (2012). Characterization of different subpopulations from bone marrow-derived mesenchymal stromal cells by alkaline phosphatase expression. Stem Cells Dev..

[2] Bowles-Welch A.C., Jimenez A.C., Stevens H.Y., Rubio D.A.F., Kippner L.E., Yeago C., Roy K. (2023). Mesenchymal stromal cells for bone trauma, defects, and disease: Considerations for manufacturing, clinical translation, and effective treatments. Bone Rep..

[3] Shelke A.R., Roscoe J.A., Morrow G.R., Colman L.K., Banerjee T.K., Kirshner J.J. (2008). Genetic alterations NIH Public Access. Bone.

[4] Steinert A.F., Rackwitz L., Gilbert F., Nöth U., Tuan R.S. (2012). Concise Review: The Clinical Application of Mesenchymal Stem Cells for Musculoskeletal Regeneration: Current Status and Perspectives. Stem Cells Transl. Med..

[5] Gimble J.M., Guilak F., Nuttall M.E., Sathishkumar S., Vidal M., Bunnell B.A. (2008). In vitro differentiation potential of mesenchymal stem cells. Transfus. Med. Hemotherapy.

[6] Vogt A., Kapetanos K., Christodoulou N., Asimakopoulos D., Birch M.A., McCaskie A.W., Khan W. (2023). The Effects of Chronological Age on the Chondrogenic Potential of Mesenchymal Stromal Cells: A Systematic Review. Int. J. Mol. Sci..

[7] Chen D., Shen J., Zhao W., Wang T., Han L., Hamilton J.L., Im H.-J. (2017). Osteoarthritis: Toward a comprehensive understanding of pathological mechanism. Bone Res..

[8] Zhu C., Wu W., Qu X. (2021). Mesenchymal stem cells in osteoarthritis therapy: A review. Am. J. Transl. Res..

[9] Kwon D.G., Kim M.K., Jeon Y.S., Nam Y.C., Park J.S., Ryu D.J. (2022). State of the Art: The Immunomodulatory Role of MSCs for Osteoarthritis. Int. J. Mol. Sci..

[10] Kempson G.E., Muir H., Swanson S.A.V., Freeman M.A.R. (1970). Correlations between stiffness and the chemical constituents of cartilage on the human femoral head. Biochim. Et Biophys. Acta (BBA)-Gen. Subj..

[11] Hauser R.A., Dolan E.E. (2011). Ligament Injury and Healing: An Overview. J. Prolotherapy.

[12] Yang G., Rothrauff B.B., Tuan R.S. (2013). Tendon and ligament regeneration and repair: Clinical relevance and developmental paradigm. Birth Defects Res. C Embryo Today.

[13] Lu V., Tennyson M., Zhang J., Khan W. (2021). Mesenchymal stem cell-derived extracellular vesicles in tendon and ligament repair—A systematic review of in vivo studies. Cells.

[14] Mao X.F., Zhang X.Q., Yao Z.Y., Mao H.J. (2024). Advances in mesenchymal stem cells therapy for tendinopathies. Chin. J. Traumatol.-Engl. Ed..

[15] Sammour I., Somashekar S., Huang J., Batlahally S., Breton M., Valasaki K., Khan A., Wu S., Young K.C. (2016). The effect of gender on mesenchymal stem cell (MSC) efficacy in neonatal hyperoxia-induced lung injury. PLoS ONE.

[16] Roman-Blas J.A., Castañeda S., Largo R., Herrero-Beaumont G. (2009). Osteoarthritis associated with estrogen deficiency. Arthritis Res. Ther..

[17] Mohamad N.V., Soelaiman I.N., Chin K.Y. (2016). A concise review of testosterone and bone health. Clin. Interv. Aging.

[18] Sozen T., Ozisik L., Calik Basaran N. (2017). An overview and management of osteoporosis. Eur. J. Rheumatol..

[19] Ji M.X., Yu Q. (2015). Primary osteoporosis in postmenopausal women. Chronic Dis. Transl. Med..

[20] Pang H., Chen S., Klyne D.M., Harrich D., Ding W., Yang S., Han F.Y. (2023). Low back pain and osteoarthritis pain: A perspective of estrogen. Bone Res..

[21] Page M.J., McKenzie J.E., Bossuyt P.M., Boutron I., Hoffmann T.C., Mulrow C.D., Shamseer L., Tetzlaff J.M., Akl E.A., Brennan S.E. (2021). The PRISMA 2020 statement: An updated guideline for reporting systematic reviews. BMJ.

[22] Page M.J., McKenzie J.E., Bossuyt P.M., Boutron I., Hoffmann T.C., Mulrow C.D., Shamseer L., Tetzlaff J.M., Akl E.A., Brennan S.E. (2021). PRISMA 2020 checklist. BMJ.

[23] Document T., OHAT Risk of Bias Rating Tool for Human and Animal Studies Organization of This Document Indirectness, Timing, and Other Factors Related to Risk of Bias 2015. pp. 1–37. https://ntp.niehs.nih.gov/sites/default/files/ntp/ohat/pubs/riskofbiastool_508.pdf.

[24] Aksu A.E., Rubin J.P., Dudas J.R., Marra K.G. (2008). Role of gender and anatomical region on induction of osteogenic differentiation of human adipose-derived stem cells. Ann. Plast. Surg..

[25] Kolliopoulos V., Tiffany A., Polanek M., Harley B.A., Harley B. (2023). Donor Variability in Human Mesenchymal Stem Cell Osteogenic Response As a Function of Passage Conditions and Donor Sex. bioRxiv.

[26] Lee H., Min S., Park J. (2019). Effects of demographic factors on adipogenic and chondrogenic differentiation in bone marrow-derived stem cells. Exp. Ther. Med..

[27] García-Álvarez F., Alegre-Aguarón E., Desportes P., Royo-Cañas M., Castiella T., Larrad L., Martínez-Lorenzo M.J. (2011). Chondrogenic differentiation in femoral bone marrow-derived mesenchymal cells (MSC) from elderly patients suffering osteoarthritis or femoral fracture. Arch. Gerontol. Geriatr..

[28] Fossett E., Khan W.S., Pastides P., Adesida A.B. (2012). The effects of ageing on proliferation potential, differentiation potential and cell surface characterisation of human mesenchymal stem cells. Curr. Stem Cell Res. Ther..

[29] Siegel G., Kluba T., Hermanutz-Klein U., Bieback K., Northoff H., Schafer R. (2013). Phenotype, donor age and gender affect function of human bone marrow-derived mesenchymal stromal cells. BMC Med..

[30] West C.C., Hardy W.R., Murray I.R., James A.W., Corselli M., Pang S., Black C., Lobo S.E., Sukhija K., Liang P. (2016). Prospective purification of perivascular presumptive mesenchymal stem cells from human adipose tissue: Process optimization and cell population metrics across a large cohort of diverse demographics. Stem Cell Res. Ther..

[31] Stölting M.N.L., Hefermehl L.J., Tremp M., Azzabi F., Sulser T., Eberli D. (2017). The role of donor age and gender in the success of human muscle precursor cell transplantation. J. Tissue Eng. Regen. Med..

[32] Riddle E.S., Bender E.L., Thalacker-Mercer A.E. (2018). Expansion capacity of human muscle progenitor cells differs by age, sex, and metabolic fuel preference. Am. J. Physiol. Cell Physiol..

[33] Reumann M.K., Linnemann C., Aspera-Werz R.H., Arnold S., Held M., Seeliger C., Nussler A.K., Ehnert S. (2018). Donor site location is critical for proliferation, stem cell capacity, and osteogenic differentiation of adipose mesenchymal stem/stromal cells: Implications for bone tissue engineering. Int. J. Mol. Sci..

[34] Barreto-Durán E., Mejía-Cruz C.C., Leal-García E., Pérez-Núñez R., Rodríguez-Pardo V.M. (2018). Impact of donor characteristics on the quality of bone marrow as a source of mesenchymal stromal cells. Am. J. Stem Cells.

[35] Scibetta A.C., Morris E.R., Liebowitz A.B., Gao X., Lu A., Philippon M.J., Huard J. (2019). Characterization of the chondrogenic and osteogenic potential of male and female human muscle-derived stem cells: Implication for stem cell therapy. J. Orthop. Res..

[36] Herrmann M., Hildebrand M., Menzel U., Fahy N., Alini M., Lang S., Benneker L., Verrier S., Stoddart M.J., Bara J.J. (2019). Phenotypic characterization of bone marrow mononuclear cells and derived stromal cell populations from human iliac crest, vertebral body and femoral head. Int. J. Mol. Sci..

[37] Mckinnirey F., Herbert B., Vesey G., McCracken S. (2021). Immune modulation via adipose derived Mesenchymal Stem cells is driven by donor sex in vitro. Sci. Rep..

[38] Lee H.J., Lee H., Na CBin Song I.S., Ryu J.J., Park J.B. (2021). Evaluation of the age-and sex-related changes of the osteogenic differentiation potentials of healthy bone marrow-derived mesenchymal stem cells. Medicina.

[39] Lee D.H., Ng J., Kim S.B., Sonn C.H., Lee K.M., Han S.B. (2015). Effect of Donor Age on the Proportion of Mesenchymal Stem Cells Derived from Anterior Cruciate Ligaments. PLoS ONE.

